# Detection and characterization of pancreatic and biliary tract cancers using cell-free DNA fragmentomics

**DOI:** 10.1186/s13046-024-03067-y

**Published:** 2024-05-15

**Authors:** Xiaohan Shi, Shiwei Guo, Qiaonan Duan, Wei Zhang, Suizhi Gao, Wei Jing, Guojuan Jiang, Xiangyu Kong, Penghao Li, Yikai Li, Chuanqi Teng, Xiaoya Xu, Sheng Chen, Baoning Nian, Zhikuan Li, Chaoliang Zhong, Xiaolu Yang, Guangyu Zhu, Yiqi Du, Dadong Zhang, Gang Jin

**Affiliations:** 1 Department of Hepatobiliary Pancreatic Surgery, Changhai Hospital, Navy Military Medical University, 168 Changhai Road, Shanghai, 200433 China; 2 Department of Clinical and Translational Medicine, 3D Medicines Inc, 158 Xin Junhuan Road, Pujiang Hi-Tech Park, Shanghai, 201114 China; 3 Department of Gastroenterology, Changhai Hospital, Navy Military Medical University, 168 Changhai Road, Shanghai, 200433 China; 4 Department of Interventional Radiology and Vascular Surgery, Zhongda Hospital, Southeast University, 87 Dingjiaqiao Road, Nanjing, Jiangsu Province 210009 China

**Keywords:** Biliopancreatic cancer, Cell-free DNA, Fragmentomics, Low-coverage whole-genome sequencing

## Abstract

**Background:**

Plasma cell-free DNA (cfDNA) fragmentomics has demonstrated significant differentiation power between cancer patients and healthy individuals, but little is known in pancreatic and biliary tract cancers. The aim of this study is to characterize the cfDNA fragmentomics in biliopancreatic cancers and develop an accurate method for cancer detection.

**Methods:**

One hundred forty-seven patients with biliopancreatic cancers and 71 non-cancer volunteers were enrolled, including 55 patients with cholangiocarcinoma, 30 with gallbladder cancer, and 62 with pancreatic cancer. Low-coverage whole-genome sequencing (median coverage: 2.9 ×) was performed on plasma cfDNA. Three cfDNA fragmentomic features, including fragment size, end motif and nucleosome footprint, were subjected to construct a stacked machine learning model for cancer detection. Integration of carbohydrate antigen 19–9 (CA19-9) was explored to improve model performance.

**Results:**

The stacked model presented robust performance for cancer detection (area under curve (AUC) of 0.978 in the training cohort, and AUC of 0.941 in the validation cohort), and remained consistent even when using extremely low-coverage sequencing depth of 0.5 × (AUC: 0.905). Besides, our method could also help differentiate biliopancreatic cancer subtypes. By integrating the stacked model and CA19-9 to generate the final detection model, a high accuracy in distinguishing biliopancreatic cancers from non-cancer samples with an AUC of 0.995 was achieved.

**Conclusions:**

Our model demonstrated ultrasensitivity of plasma cfDNA fragementomics in detecting biliopancreatic cancers, fulfilling the unmet accuracy of widely-used serum biomarker CA19-9, and provided an affordable way for accurate noninvasive biliopancreatic cancer screening in clinical practice.

**Supplementary Information:**

The online version contains supplementary material available at 10.1186/s13046-024-03067-y.

## Background

Biliopancreatic cancers including cholangiocarcinoma (CCA), gallbladder cancer (GBC), and pancreatic cancer (PAC) are a group of malignancies characterized by extremely poor prognosis, which mainly attributes to late diagnosis and limited therapeutic intervention [1, 2]. To better manage these diseases and improve patient survival, accurate detection, especially early detection, is of critical importance. With the rapid development of liquid biopsy, cell-free DNA (cfDNA) has provided a noninvasive approach for the diagnosis of solid malignancies [3]. Normally, cfDNA originates mostly from hematopoietic cells, while in cancer patients, a fraction of cfDNA molecules could be released from the malignant cells [4]. Previous studies have demonstrated that plasma cfDNA carried genetic and epigenetic information of their tissue of origin [5, 6]. However, due to the low concentration of tumor-related cfDNA in plasma, detectable variations are limited among patients, especially at early stages [7]. To improve the practicality of cfDNA, recent research has focused more on its detailed physical and molecular features, which held great promise for clinical application [8].

Plasma cfDNA fragmentomics is an emerging field that covers various features like fragment size, end point and nucleosome footprint, where many studies have demonstrated that significant difference could be observed between cancer patients and healthy individuals [9–12], even at early stages [13–16]. Cristiano et al. introduced an approach focusing on the fragmentation size ratio in a multi-cancer cohort, in which the machine learning model had sensitivities of detection ranging from 57% to > 99% among seven cancer types at 98% specificity, with an overall AUC of 0.94 [9]. The fragment size feature based models were also reported in the early detection of primary liver cancer which showed excellent sensitivities in detecting early-stage cancer (I: 95.9%, II: 97.9%) and small tumor (≤ 3 cm: 98.2%) [15]. Besides, the end motif feature was widely studied for construction of stacked machine learning models in the early detection of multiple cancers such as colorectal adenocarcinoma and lung adenocarcinoma [14, 16]. Since the genomic distribution of nucleosomes was considered to be cell-type specific [17], nucleosome footprint was found to be another important feature that could inform the tissues contributing to cfDNA. A recent study on hepatocellular carcinoma has revealed that nucleosome footprint was the best individual diagnostic feature for differentiating hepatocellular carcinoma from liver cirrhosis in both validation (AUC = 0.971) and test sets (AUC = 0.973) [13]. However, among biliopancreatic cancers, detection models based on these fragmentomic features still remain less investigated.

In this study, we constructed a fragmentomics-based machine learning model for detecting the closely located biliopancreatic cancers including CCA, GBC, and PAC using low-coverage whole-genome sequencing data. The stacked model by fragment size, end motif, and nucleosome footprint showed excellent performance in detecting biliopancreatic cancers, which exhibited higher sensitivity and specificity when integrated with carbohydrate antigen 19–9 (CA19-9). Moreover, our method demonstrated the potential utility of cfDNA fragmentomics in differentiating biliopancreatic cancer subtypes.

## Methods

### Patients enrollment and sample collection

Patients with treatment-naïve clinically diagnosed biliopancreatic malignancies were collected from Changhai prospective database (Changhai Hospital, Shanghai, China). Blood samples were collected before surgery or biopsy for cfDNA extraction and CA19-9 examination (Roche Diagnostics GmbH, 11,776,193: 39 IU/mL as cut off value). Exclusion criteria were as follows: (1) Patients without biliopancreatic cancers confirmed by pathological examination. (2) Patients with inconclusive pathology results. (3) Patients simultaneously suffering from other tumors. (4) Blood specimens with hemolysis levels > grade 4. Patients were staged according to the 8th edition of the American Joint Committee of Cancer (AJCC) tumor-node-metastasis (TNM) staging system. All participants provided signed written informed consent to use their blood samples and clinical data, and were divided randomly into a training cohort and a validation cohort. This study was conducted in accordance with the national guideline and approved by the Ethics Committee of Changhai Hospital (CHEC2018-112).

### Plasma cfDNA extraction

Blood samples were collected in BEAVER Cell Free DNA Tubes (Beaver, 43,803). To harvest plasma, the blood samples were centrifuged at 1600 g for 10 min at 4 °C, after which the hemolysis level was determined and recorded. The samples with hemolysis level ≤ grade 3 were used for further experiments. The collected supernatant was centrifuged at 16,000 g for 15 min at 4 °C to remove the cell debris. Then, the supernatant was stored in 1 mL aliquots at − 80 °C prior to DNA extraction. Plasma samples were thawed in a 37 °C water bath and centrifuged at 1600 g for 10 min at 4 °C. QIAamp Nucleic Acid Kit (Qiagen) was used to isolate plasma cfDNA. Qubit 3.0 fluorometer (Thermo Fisher) and Agilent 2100 bioanalyzer (Agilent Technologies Inc.) were used to detect the cfDNA concentration, purity, and fragment distributions.

### Plasma cfDNA library construction and low-coverage WGS

The qualified cfDNA samples were used for cfDNA library construction followed by low-coverage WGS. The library was prepared using KAPA DNA Hyper Prep Kit (KAPA, KK8504) in accordance with the manufacturer’s instructions. The input amount of each cfDNA sample was 10 ng. The base end was replenished, and an “A” tail was added. Then, the joint was connected and purified, and seven circulating enrichment libraries were amplified by PCR. After purification and elution in 25 µL of eluent, the plasma cfDNA library concentration and the fragment distribution were determined by Qubit (Thermo Fisher) and Agilent 2100 bioanalyzer (Agilent Technologies), respectively. NovaSeq 6000 platform (Illumina) was used for WGS with a sequencing strategy of 2 × 150 bp and sequencing volume of ~ 10 G (~ 3 ×).

### Sequencing alignment and quality control

The raw sequencing data were trimmed by Trimmomatic as part of the quality control protocol. The qualified reads were then mapped onto the human reference genome (ftp://ftptrace.ncbi.nih.gov/1000genomes/ftp/technical/reference/human_g1k_v37.fasta.gz) using the sequence aligner BWA after PCR duplicates were removed by Picard toolkit (http://broadinstitute.github.io/picard/). The final coverage depths for these samples ranged from 1.9 × to 4.4 × (median coverage 2.8 ×).

### Data processing

After removing adapters, the sequencing data in FASTQ format were aligned to the hg19 reference genome. Low-quality and repetitive reads were removed. Only reads meeting the following criteria were kept: (1) Aligned to autosomes; (2) Quality score greater than 20; (3) Insertion size between 150 and 600 bp; (4) Properly paired; (5) Reference region without degenerate bases.

### Identifying fragmentomics features

Following the approach called “DNA evaluation of fragments for early interception” (DELFI) method [9], the genome was divided into 504 5-Mb bins. Coverage of short and long fragments in each bin was calculated, where short fragments were defined as those with a length [130,177] and long fragments were those with a length [177, 237]. The z-score standardized short and total (short and long) fragment coverage in the 504 bins was used as the input for machine learning with 1008 features in total.

For end motif features, we defined the 6th bp on the 5’ end of a cfDNA fragment as the 1st position due to our unique molecular identifier (UMI)-attached sequence data. UMI is commonly introduced into sequencing to increase accuracy, while we found that they affected the frequencies of end motifs. Comparison of samples with and without UMIs showed that the motif frequencies of UMI attached samples could match those of UMI-unattached samples in previous studies when counting from the 6th position rather than the 1st position (Figure S1). 3-bp motifs from the 1st, 4th, and 7th positions of the 5’ end of the cfDNA fragments were counted for each sample. One 3-bp motif has 64 different combinations. Their frequencies were combined into a 9-bp codon motif with 192 (64 × 3) features.

To compute nucleosome footprint features, we defined the central region of a gene as ± 250 bp around transcription start site (TSS) and the reference region of the gene as the sum of the upstream [− 2000, − 1000] bp region and the downstream [1000, 2000] bp region around its TSS. The nucleosome footprint of a gene was then defined as the mean coverage of the central region divided by the mean coverage of the reference region. A total of 24,639 genes were selected as features based on their nucleosome footprint profiles across samples.

### Machine learning

Ten-fold cross-validation on the training cohort was used to select models and optimize criteria. The cutoff was selected as the value that minimized Gini impurity in the training cohort. LinearSVC was selected as the machine learning model for fragment size and nucleosome footprint features. RandomForest was selected as the model for end motif features. A stacked model trained on the three cfDNA features were built using stacking learning with logistic regression as the meta-learner. When training models for a specific cancer type, only non-cancer samples and samples of the specific cancer type were used.

To construct the CF (CA19-9 and fragmentomics) model which is based on CA19-9 and stacked model scores, log_2_(x + 1) transformation of CA19-9 and ten-fold cross-validated estimates of the stacked model were computed in the training cohort as input scores. LinearSVC was selected as the machine learning classifier for the final model.

### Statistics

The *P*-values of the risk scores were computed using the Mann–Whitney U (Mann–Whitney U test) function in the Python SciPy package. The heatmaps were constructed using the Python Seaborn package (https://seaborn.pydata.org/citing.html). *P*-values less than 0.05 were considered to be statistically significant.

### Ethics statement

The study was conducted in accordance with the Declaration of Helsinki. This study was approved by the Ethics Committee of Changhai Hospital (CHEC2018-112). All participants provided signed written informed consent to use their blood samples and clinical data.

### Role of the funding source

The sponsors did not have any role in the study design, data collection, data analysis, interpretation, or writing of the manuscript.

## Results

### Participant characteristics in the cohorts

**Fig. 1:**
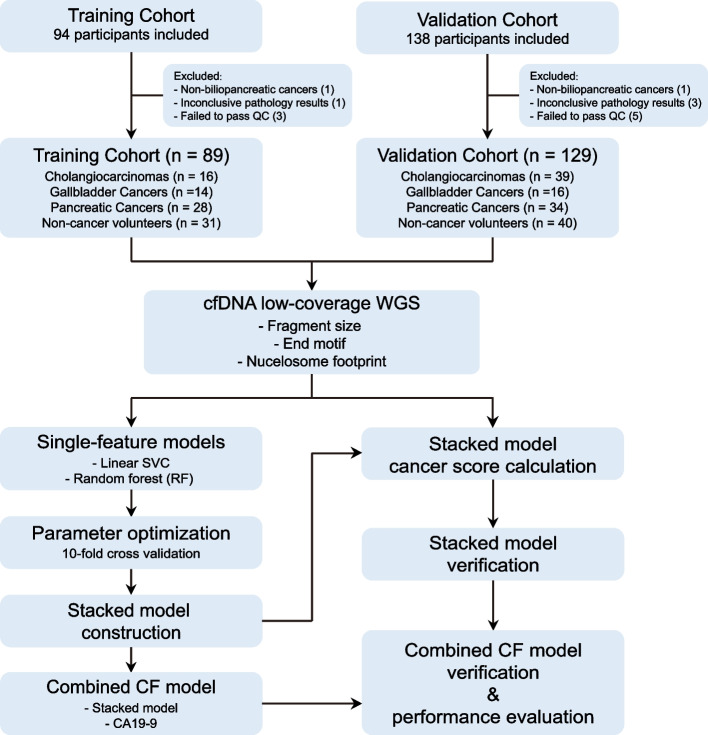
Study design. The training cohort (*n* = 89) included 16 cholangiocarcinoma (CCA), 14 gallbladder cancer (GBC), 28 pancreatic cancer (PAC) patients and 31 non-cancer volunteers. The validation cohort (*n* = 129) included 39 CCA, 16 GBC, 34 PAC patients and 40 non-cancer volunteers. Blood samples were collected from biliopancreatic cancer patients and non-cancer individuals. cfDNA was extracted from plasma samples, subjected to low-coverage WGS, and analyzed to determine cfDNA fragmentomics features across the genome. Three different features, including fragment size, end motif, and nucleosome footprint, were calculated to build detection models using the LinearSVC algorithm or the RandomForest algorithm with tenfold cross-validation. The single-feature models were then assembled into a large matrix to train the stacked model. CA19-9 was integrated with cfDNA fragmentomics features to generate the final CA19-9 and fragmentomics for cancer detection (CF) model

A total of 232 participants were recruited in this study, including 73 non-cancer volunteers and 159 patients clinically diagnosed with biliopancreatic cancers (Fig. 1). After excluding two patients who did not have pathologic biliopancreatic cancers, four with inconclusive pathology results, and eight who did not meet the quality control standards, 147 patients and 71 non-cancer volunteers finally remained. The training cohort consisted of 89 individuals, including 58 patients (16 with CCA, 14 with GBC, 28 with PAC) and 31 non-cancer volunteers. The validation cohort contained 129 individuals, including 89 patients (39 with CCA, 16 with GBC, 34 with PAC) and 40 non-cancer volunteers. The clinical characteristics of the patients are shown in Table 1. Blood samples were obtained before surgery, and plasma cfDNA was subjected to low-coverage WGS. The average coverage depth was 2.9 × (Range: 1.9 × –4.4 ×).

**Table 1:** Participant characteristics

		Cohort		Training (*n* = 89)	Validation (*n* = 129)
**Non-cancer**		Total, n (%)		31 (34.8%)	40 (31.0%)
		Female, n (%)		12 (38.7%)	12 (30.0%)
**Cancer**	**All**	Total, n (%)		58 (65.2%)	89 (69.0%)
		Female, n (%)		25 (43.1%)	24 (27.0%)
		Age, mean ± SD		62.7 ± 10.2	62.2 ± 10.0
		Stage TNM, n (%)	I	10 (17.2%)	9 (10.1%)
			II	21 (36.2%)	39 (43.8%)
			III	21 (36.2%)	29 (32.6%)
			IV	6 (10.3%)	12 (13.5%)
		CA19-9 (U/ml), n (%)	≤ 39	18 (31.0%)	19 (21.3%)
			> 39	40 (69.0%)	70 (78.7%)
	**PAC**	Total, n (%)		28 (31.5%)	34 (26.4%)
		Female, n (%)		14 (50.0%)	8 (23.5%)
		Age, mean ± SD		67.5 ± 7.1	65.0 ± 9.6
		Stage TNM, n (%)	I	8 (28.6%)	8 (23.5%)
			II	13 (46.4%)	18 (20.6%)
			III	3 (10.7%)	7 (23.5%)
			IV	4 (14.3%)	1 (2.9%)
		Tumor size (cm), n (%)	< 3	9 (32.1%)	14 (41.2%)
			3–5	15 (53.6%)	13 (38.2%)
			> 5	4 (14.3%)	7 (20.6%)
		CA19-9 (U/ml), n (%)	≤ 39	7 (25.0%)	5 (14.7%)
			> 39	21 (75.0%)	29 (85.3%)
	**GBC**	Total, n (%)		14 (15.7%)	16 (12.4%)
		Female, n (%)		7 (50.0%)	7 (43.8%)
		Age, mean ± SD		59.1 ± 10.9	58.9 ± 10.1
		Stage TNM, n (%)	I	0 (0%)	0 (0%)
			II	0 (0%)	1 (6.2%)
			III	12 (85.7%)	8 (50.0%)
			IV	2 (14.3%)	7 (43.8%)
		Tumor size (cm), n (%)	< 3	2 (14.3%)	4 (25.0%)
			3–5	9 (64.3%)	7 (43.8%)
			> 5	3 (21.4%)	5 (31.2%)
		CA19-9 (U/ml), n (%)	≤ 39	3 (21.4%)	4 (25.0%)
			> 39	11 (78.6%)	12 (75.0%)
	**CCA**	Total, n (%)		16 (18.0%)	39 (30.2%)
		Female, n (%)		4 (25.0%)	9 (23.1%)
		Age, mean ± SD		57.4 ± 9.7	61.0 ± 9.5
		Stage TNM, n (%)	I	2 (12.5%)	1 (2.6%)
			II	8 (50.0%)	20 (51.3%)
			III	6 (37.5%)	14 (35.9%)
			IV	0 (0%)	4 (10.2%)
		Tumor size (cm), n (%)	< 3	9 (56.2%)	28 (71.8%)
			3–5	6 (37.5%)	9 (23.1%)
			> 5	1 (6.2%)	2 (5.1%)
		CA19-9 (U/ml), n (%)	≤ 39	8 (50.0%)	10 (25.6%)
			> 39	8 (50.0%)	29 (74.4%)

### Identifying cfDNA fragmentomics features

**Fig. 2:**
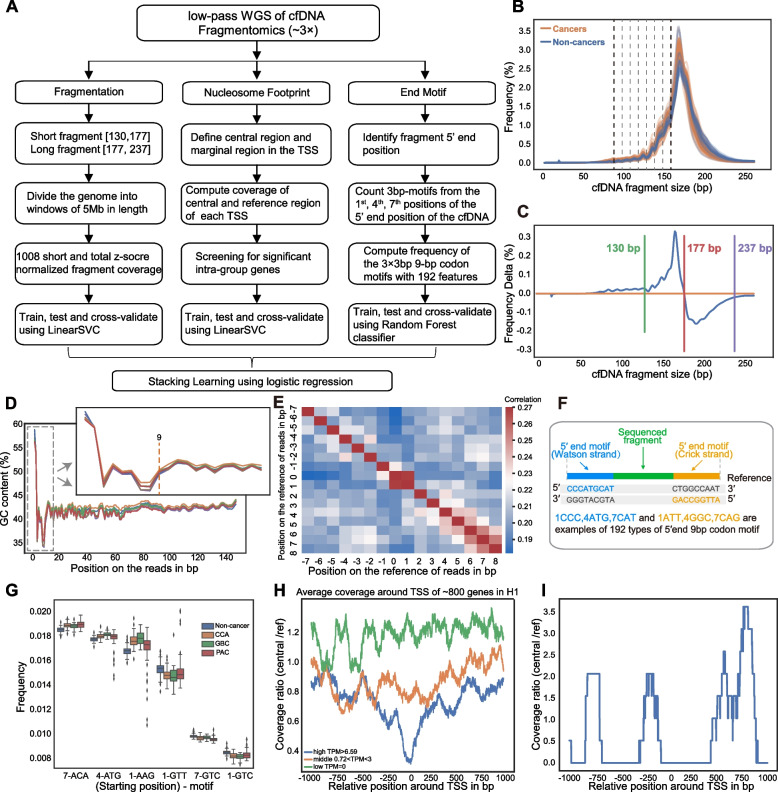
Identification of cfDNA fragmentomics features. **A** Flowchart for model construction. **B** Fragment size frequency distributions of 89 plasma samples from 58 cancer patients and 31 non-cancer individuals of the training cohort in percentage. **C** The average fragment size frequency distribution of the cancer samples subtracted by that of the non-cancer samples. **D** The GC content of 140-bp cfDNA reads from the 5’ end. The locally enlarged figure shows the first 20 bp. **E** The correlation matrix of occurrence frequencies of nucleic acid bases around the 5’ end of the cfDNAs on the reference genome. Zero is the starting base of each cfDNA fragment. **F** Schematic illustration of the determination of plasma DNA 9-bp (3 × 3-bp) code end motifs. **G** Box plot of six representative motifs showing differential frequencies between non-cancer and cancer patients in the training cohort. **H** Average plasma DNA coverage patterns around the transcription start sites (TSSs) of genes with varied expression levels in a non-cancer individual (green, TPM = 0; orange, TPM > 0.72 and < 3; blue, TPM > 6.6). **I** Plasma DNA coverage patterns around the TSS of one specific gene in a non-cancer individual. TPM, transcripts per million

We extracted cfDNA features including fragment size, end motif, and nucleosome footprint from the WGS data following the workflow shown in Fig. 2A. Consistent with previous studies, our training cohort showed that the modal size was approximately 166 bp which was related to nucleosomal structure [18], and the fragment size distribution had a series of successive peaks about 10 bp in the 90–160 bp range, and compared to non-cancers, the concentration of fractional plasma cfDNA in cancers increased, resulting in the size profile of plasma cfDNA shifting toward the left (Fig. 2B-C) [12, 19]. Following the DELFI approach, we defined short fragments with a length [130, 177] and long fragments with a length [177, 237], and used z-score standardized short and total (short and long) fragment coverage as features.

For end motif features, 4-bp end motif has been studied most [13, 20], while others proposed that 6-bp end motif might be better [14]. After examining the GC content from the 5’ end of the cfDNA fragments, we found that its deviation from the mean extended up to 9 bp (Fig. 2D), which indicated that a 9-bp end motif could provide more information. However, a direct 9-bp motif would develop numerous combinations. To simplify, we then examined the correlations between base occurrences around the starting positions of the cfDNA fragments on the reference genome and found that they were more likely to be correlated in adjacent positions (Fig. 2E). Therefore, we divided the 9-bp motif into three 3-bp motifs with a total of 192 features (Fig. 2F). Figure 2G shows six representative motifs with significantly different frequencies between cancer patients and non-cancer individuals. Three motifs (1-GTT, 7-GTC, and 1-GTC) were more concentrated in non-cancer individuals, while the other three (7-ACA, 4-ATG, and 1-AAG) showed higher frequencies in cancer patients. We found that the 9-bp codon motif performed better than the more commonly used 4-bp motif in the training cohort (Figure S2).

In 2016, Ulz et al. developed a computational approach for whole-genome sequencing of plasma DNA called nucleosome footprint to infer gene expression [21]. By identifying two discrete regions at TSSs, a 2000-bp region centered on the TSS (2 K-TSS coverage) and a − 150 bp to + 50 bp region with respect to the TSS (NDR coverage), nucleosome occupancy results in different read depth coverage patterns for expressed and silent genes [21]. To examine whether the nucleosome footprint in our study could inform gene expression, we compared the calculated the average DNA coverage patterns around gene TSSs to the expression profile of non-cancer individuals from public database (GTEx) [22], which showed that nucleosome depletion around TSS was closely associated with gene expression (Fig. 2H-I).

### Machine learning models construction for biliopancreatic cancers

**Fig. 3:**
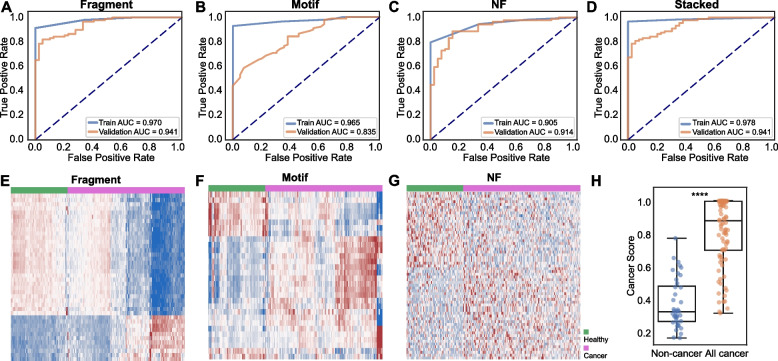
Construction and evaluation of machine learning models for cancer detection using cfDNA fragmentomics features. **A-D** The receiver operating characteristic (ROC) curves of the training and validation cohorts to evaluate the performance of the models built on different cfDNA features (fragment size (**A**), end motif (**B**), nucleosome footprint (**C**), stacked features (**D**)) using machine learning algorithms in distinguishing patients with biliopancreatic cancer from non-cancer individuals. **E–G** Heat map visualization of the cfDNA fragmentomics features including fragment (**E**), motif (**F**), and nucleosome footprint (**G**) for cancer patients or non-cancer individuals. **H** Box plots illustrating cancer scores calculated from the stacked model in the non-cancer and cancer groups in the validation cohort. ****, *P* < 0.0001

Diagnostic models for biliopancreatic cancers were constructed based on the cfDNA fragment size (Fragment, Fig. 3A), end motif (Motif, Fig. 3B), and nucleosome footprint (NF, Fig. 3C) alone or in combination (Stacked, Fig. 3D). The area under curve (AUC) values of the fragment size, the end motif, the nucleosome footprint, and the stacked models in the training cohort were 0.970, 0.965, 0.910, and 0.983, respectively, and their AUCs in the validation cohort were 0.941, 0.835, 0.914, and 0.941, respectively (Fig. 3A-D, Table S1, Table S2), which demonstrated that the stacked model was superior to the three single feature models in distinguishing biliopancreatic cancer patients from non-cancer volunteers. To optimize model performance and avoid overfitting, ten-fold cross-validation was performed based on the training cohort (Fragment AUC: 0.970, 95% CI: 0.889–1.000; Motif AUC: 0.965, 95% CI: 0.889 − 1.000; NF AUC: 0.905, 95% CI: 0.833 − 1.000; stacked AUC: 0.978, 95% CI: 0.944 − 1.000) (Table S3). Heat map visualizations of the fragment size (Fig. 3E), the end motif (Fig. 3F), and the nucleosome footprint feature matrices (Fig. 3G) exhibited clear separation between non-cancer volunteers and cancer patients. Cancer scores were calculated from the stacked model, with the scores being significantly higher in cancer patients than in non-cancer volunteers (Fig. 3H).

We then used lower coverage WGS data to explore the limit of detection for the machine learning model. The WGS data was downsampled to 1 × and 0.5 × coverage before training the model using one feature alone or three features in combination. Although the performance of the stacked model gradually decreased with the lowering of the sequencing depth, the AUC trained on 0.5 × data still remained 0.905 in the validation cohort (Figure S3A). Among the models based on single features, we found that the fragment size models were affected least by downsampling (0.5 × data AUC: 0.903), while the NF models were affected most (0.5 × data AUC: 0.619) (Figure S3B-D), which was consistent with previous studies that the performance of NF relied on the sequencing depth [6, 23]. The sensitivities at 95.0% specificity of the 1 × model and 0.5 × model also slightly decreased compared with the original model (1 × model: 0.742, 0.5 × model: 0.708, original model: 0.809) (Table S4). Taken together, these results suggested that our model showed good performance for biliopancreatic detection even when using extremely low-coverage WGS data (0.5 ×).

### Model differentiating biliopancreatic cancer subtypes

**Fig. 4:**
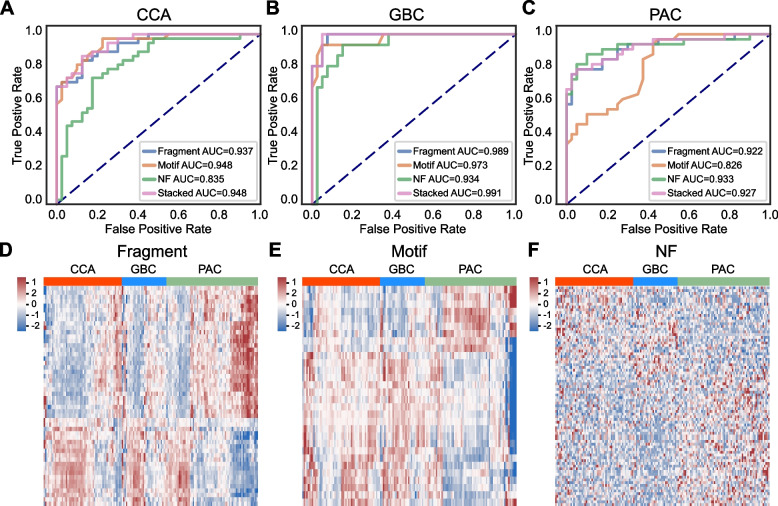
Contributions of the detection models using cfDNA fragmentomics features for each biliopancreatic cancer type. **A-C** ROC curves showing the performance of the models trained on each cfDNA feature alone or stacked of the CCA (**A**), GBC (**B**), and PAC (**C**) validation cohorts. **D-F** Heat map visualization of the cfDNA fragmentation features including fragment size (**D**), motif (**E**), and nucleosome footprint (**F**) of CCA, GBC, and PAC samples

We first evaluated the performance of the machine learning models on each subtype (Fig. 4A-C, Table S2). The AUCs of the fragment size models were relatively stable among all three cancer types, reaching 0.937 for CCA, 0.989 for GBC and 0.922 for PAC in the validation cohort, while the classification powers of end motif models and nucleosome footprint models were not even among different subtypes (End motif model AUCs: 0.948 for CCA, 0.973 for GBC and 0.826 for PAC in the validation cohort; Nucleosome footprint model AUCs: 0.835 for CCA, 0.934 for GBC and 0.933 for PAC in the validation cohort). The stacked models trained on CCA, GBC, and PAC samples had an AUC of 0.948, 0.991, and 0.927 in the validation cohort, respectively. Heat maps of each cfDNA feature revealed distinctive patterns among the three cancer subtypes (Fig. 4D-F), suggesting that they might contribute to identifying the tissue of origin of cancers.

To examine whether the cfDNA features could provide differentiation of biliopancreatic cancer subtypes, we then trained independent models on one feature alone or three features in combination (Figure S4). Among all of the models, we found that the nucleosome footprint feature exhibited the highest AUC in distinguishing PAC from CCA or/and GBC (AUC: 0.750 for CCA, 0.686 for GBC, 0.755 for CCA and GBC), while the fragment size, end motif, or stacked models showed unsatisfying performance in distinguishing different cancer subtypes, which indicated that nucleosome footprint might be the most helpful feature for inferring tissue of origin.

Next, we try to further explore the role of nucleosome footprint on differentiating subtypes. The gene coefficients were extracted from the NF model (Table S5), and the top 500 genes that contribute to predicting PAC against CCA/GBC were selected for enrichment analysis in ToppGene [24]. The enrichment analysis showed that the selected genes were significantly enriched in “olfactory receptor activity” (GO MF), “OLFACTORY SIGNALING PATHWAY” (Reactome Pathways), and “OLFACTORY TRANSDUCTION” (KEGG Pathways) (Table S6). Olfactory receptors were reported to be highly expressed in the pancreas and regulate insulin secretion [25], and the olfactory transduction pathway was found as one of the most significant pathways associated with risk of pancreatic cancer [26]. Our results suggested that the activity of this pathway was discernable in the cfDNA nucleosome footprint and helped to differentiate PAC from CCA/GBC.

### Cancer scores of machine learning models and clinical characteristics

**Fig. 5:**
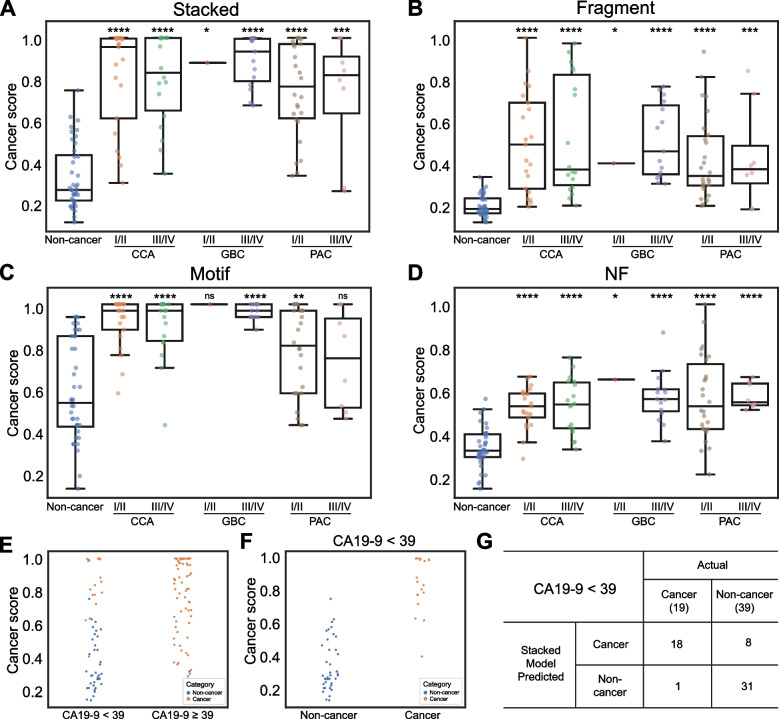
Correlation analysis between cancer scores and clinical characteristics of patients with biliopancreatic cancer. **A-D** The patients were separated into two groups, stage I/II and stage III/IV, for each cancer type, and the cancer scores of the stacked model (**A**), the fragment size model (**B**), the end motif model (**C**), and the NF model (**D**) were calculated and compared with non-cancer volunteers. **E** Comparison of cancer scores of the stacked model in CA19-9 positive (CA19-9 ≥ 39 IU/mL) or negative (CA19-9 < 39 IU/mL) patients. **F** Comparison of cancer scores between non-cancer and cancer patients in the CA19-9–negative group. **G** Patient classification of the stacked model in the CA19-9–negative group. *, *P* < 0.05; **, *P* < 0.01; ***, *P* < 0.001; ****, *P* < 0.0001; ns, no significance

Cancer scores were computed for the samples in the validation cohort using either the stacked model or the models trained on each cfDNA feature. As shown in Fig. 5A-D, the cancer scores of the Stacked model, the Fragment model, the Motif model, and the NF model were significantly higher in the cancer groups than in the non-cancer group. Meanwhile, it should be noted that there was no significant difference in cancer scores between early and late clinical stages among the three cancer types, suggesting that these models have similar performance in detecting both early- and late-stage biliopancreatic cancers.

Since CA19-9 is a commonly used biomarker for biliopancreatic patients in clinical practice [27], we then examined the performance of cancer score from the Stacked model and CA19-9 in detecting cancer samples. The positive rates of CA19-9 (> 39 U/mL) in the training and validation cohort were 69.0% and 78.7% for all patients, 50.0% and 74.4% for CCA patients, 78.6% and 75.0% for GBC patients, and 75.0% and 85.3% for PAC patients (Table 1). We grouped the participants in the validation cohort into a CA19-9 negative group and a CA19-9 positive group and found that the cancer score of the CA19-9 positive group was significantly higher than that of the CA19-9 negative group (*P* < 0.0001) (Fig. 5E). According to the CA19-9 classification, there was one non-cancer sample in the CA19-9 positive group, while a significant proportion of the samples in the CA19-9 negative group (32.8%, 19/58) were actual cancer patients, suggesting the high specificity but low sensitivity of CA19-9. To meet this striking deficiency, we investigated the diagnostic ability of cancer score from the Stacked model in the CA19-9 negative group. As shown in Fig. 5F, the cancer scores of patients were significantly higher than that of non-cancer volunteers in the CA19-9 negative group (*P* < 0.0001). Furthermore, we found that, in the CA19-9 negative group, the cancer score from the Stacked model achieved high sensitivity (18 out of 19, 94.7%) in differentiating cancer samples from non-cancer samples (Fig. 5G). These results suggested that cancer score generated by the cfDNA fragmentomics model could be an important complement to CA19-9 in distinguishing biliopancreatic cancers from non-cancer samples.

### Construction of the final CF model integrating cfDNA fragmentomics and CA19-9

**Fig. 6:**
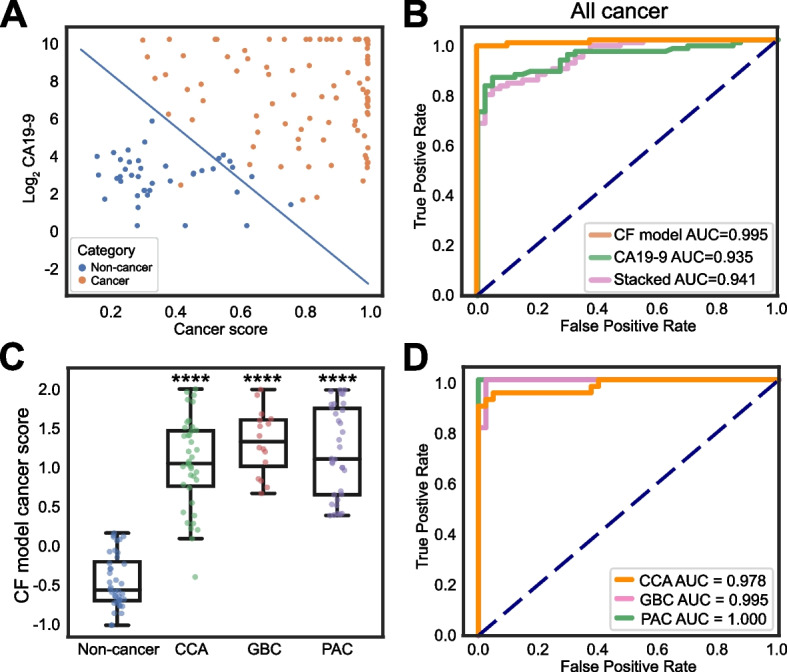
Performance evaluation of the CF model. **A** The LinearSVC classifier visualized as a single line in all cancer patients in the validation cohort. **B** ROC curve of the stacked model (pink), CA19-9 model (green) and CF model (orange) in the validation cohort. **C** Comparison of cancer scores calculated from the CF model in each biliopancreatic cancer type. **D** ROC curves of the CF model generated for each biliopancreatic cancer type. ****, *P* < 0.0001

According to above, we wondered whether the integration of CA19-9 and the stacked model would help optimize the detection of biliopancreatic cancers. We then trained a LinearSVC classifier as the CA19-9 and fragmentomics (CF) model on the training cohort using the cancer scores from the Stacked model and the log2-transformed CA19-9 values as the features. Visualized as a single line in the two-dimensional feature space, the classifier separated the cancer and non-cancer samples well (Figure S5A) and performed excellently with an AUC of 0.982 and sensitivity of 97.8% at 95.0% specificity in the training cohort (Figure S5B). Next, we applied the trained classifier to the validation cohort, and similar performance was observed with an AUC of 0.995 and sensitivity of 97.8% at 95.0% specificity (Fig. 6A-B, Table S7). Cancer scores were also calculated from the CF model, which were significantly elevated in all cancer patients (Figure S5C) and in each biliopancreatic cancer type (Fig. 6C, Figure S5D) compared with non-cancer individuals. We then tested similar models on biliopancreatic cancer subtypes, which showed AUC of 0.978 and sensitivity of 94.9% at 95% specificity for CCA, AUC of 0.995 and sensitivity of 100% at 95% specificity for GBC and AUC of 1.000 and 100% at 95% specificity for PAC in the validation cohort (Fig. 6D, Figure S5E-M, Table S7). These results supported that our integrated predictive model with CA19-9 and cfDNA fragmentomics features demonstrated an excellent detection ability for biliopancreatic cancers, shedding light on more accurate noninvasive biliopancreatic cancer screening in clinical practice.

## Discussion

Liquid biopsy, particularly cfDNA, has been widely studied to facilitate noninvasive cancer screening for improve patient prognosis [8]. With recent advancement in sophisticated technologies such as machine learning, cfDNA fragmentomics exhibited great potential for distinguishing cancer-derived cfDNA and determining tissue of origin [28]. Among the most common malignancies like liver cancer, colorectal adenocarcinoma and lung adenocarcinoma, machine learning models based on cfDNA fragmentomics have been well established to provide accurate assays for cancer detection and early screening [14–16, 29]. However, for the biliopancreatic cancers with very dismal outcomes, the characteristics of cfDNA fragmentomics and its performance on cancer diagnostics remains largely unknown. To the best of our knowledge, this study provided the first systematic characterization of cfDNA fragmentomics in biliopancreatic cancers. We investigated the potential application of cfDNA fragmentomics in detection biliopancreatic cancers, revealing that the ensemble stacked machine learning model of fragment size, end motif and nucleosome footprint showed high prediction accuracy in differentiating biliopancreatic cancer patients from healthy volunteers, which performed better when combined with CA19-9. Besides, our method could also be used to distinguish biliopancreatic cancer subtypes. This study offered valuable support for the clinical application of cfDNA fragmentomics in improving diagnostics of biliopancreatic cancers.

The fact that cfDNA has a very short half-life (~ 2 h) makes it an ideal biomarker, which carries the genetic and epigenetic information of its origin tissues reflecting the current state of a disease [28]. We previously demonstrated that preoperative detection of main driver mutations in the plasma cfDNA could be used to inform the prognosis of resectable PAC patients and help in optimizing surgical selection, but this requires prior knowledge of critical alterations in tumors [30]. Our recent research on cfDNA methylation signature found that genome-wide methylation profiles provided potential utility for noninvasive detection of early PAC [31]. Moreover, methylation-based approaches has the advantages over detection of mutations to detect organ-specific cfDNA fragments by reducing false-positive test results due to clonal haematopoiesis [32]. Recently, Ben-Ami and colleagues have proved that the combination of 9-loci cfDNA methylation panel, CA19-9 and serum protein marker TIMP1 exhibited greater discrimination of early stage PDAC than CA19-9 alone [33], and Hartwig et al*.* developed a methylation biomarker panel with better greater discrimination power for pancreato-biliary cancers than CA19-9 [34]. However, high cost and complexity as well as the relatively small number of detectable epigenetic alterations could be the limitations for further clinical application [31]. To increase the sensitivity of cancer detection with cfDNA, many genome-wide approaches for analysis of cfDNA fragmentation profiles has been developed, which exhibited the potential to identify a large number of tumor-derived changes in the circulation. For PAC, it has been reported that, compared to healthy control, plasma cfDNA had significantly shorter fragment size and higher concentration in patient samples, which were associated with worse outcomes [35]. Similar results could also be observed in CCA and GBC that the concentration of plasma cfDNA in patient samples were markedly higher than that in non-cancer controls and increased with tumor stage or tumor size [36, 37]. In this study, we found that fragment size was a powerful biomarker for differentiating biliopancreatic cancers from healthy volunteers. Besides, end motif and nucleosome footprint were identified as another two critical features for biliopancreatic cancers detection. Furthermore, the stacked model based on these fragmentomics features exhibited excellent sensitivity and specificity in biliopancreatic cancers diagnostics, even at extremely low sequencing depth of 0.5 × , providing new insights for revolutionizing blood-based cancer detection.

Since nucleosome occupancy patterns are different among various tissues [12], it is possible to use cfDNA fragmentation patterns to infer the characteristics of the epigenome for determining its tissue origin. Based on fragment size feature alone, DELFI approach has demonstrated the potential to distinguish between seven different cancers, which had a 61% accuracy (95% CI 53% − 67%) that increased to 75% (95% CI 69% − 81%) when assigning ctDNA to one of two sites of origin [9]. Later, a multidimensional model developed by Bao et al. based on five distinct fragmentomics features covering cfDNA fragmentation size, motif sequence and copy number variation showed promising results in detecting cancers from distant anatomical locations (97.4%, 94.3% and 85.6% for primary liver cancer, colorectal adenocarcinoma and lung adenocarcinoma, respectively) [16]. In addition, cfDNA fragmentomics could also be used to classify cancer subtypes. A recent study on cfDNA fragmentomics of primary liver cancer revealed that the fragmentomics-based machine learning model showed potential for distinguishing intrahepatic cholangiocarcinoma from hepatocellular carcinoma (AUC: 0.776) [15]. In this study, we focused on the three closely located biliopancreatic cancers including CCA, GBC and PAC, which could be more difficult in differentiating due to similar development backgrounds. As expected, differentiation between CCA and GBC was not satisfying by either one fragmentomics feature or stacked model, while it was easier to differentiate PAC from biliary tract cancers (CCA and GBC) (Figure S4). Particularly, we noticed that the patterns of NF carried more cell-type-specific information compared to fragment size and end motif, which was consistent with other studies [38]. According to the gene set enrichment analysis of top 500 genes that contribute to predicting PAC against CCA/GBC, olfactory related pathways were found most significantly enriched. It has been reported that in addition to the olfactory system, olfactory receptors were also expressed in other human tissues like pancreas and might play a crucial role in the initiation of different cancers [39]. In pancreatic cancers, many somatic mutations were found in olfactory receptor genes [40], and gene expression in the olfactory pathway were mostly significantly affected in pancreatic cancer [41]. Moreover, the differentially methylated and expressed genes of pancreatic cancer were also found mainly related to olfactory transduction [42]. With these results, our study provided important evidence to confirm the potential utility of cfDNA fragmentomics in identifying the tissue of origin.

As the most widely used biomarker in biliopancreatic cancers, CA19-9 (also called sialyl Lewis A antigen) plays an indispensable role in cancer diagnosis and prognostic prediction [43]. However, CA19-9 is not ideal. In addition to false positive results caused by biliary tract obstruction and inflammation, pancreatitis and other digestive cancers, false negative results that Lewis antigen negative individuals, occurring in 5% to 10% of the population, have very low or even absent secretion of CA19-9 could be the major shortfall for its clinical application [44]. Therefore, the development of new biomarkers to assist CA19–9 in biliopancreatic cancers is highly necessary. Previous studies have proposed that traditional tumor biomarkers carcinoembryonic antigen (CEA) and CA125 had the potential to be applied in Lewis negative patients with pancreatic cancer [44]. Our recent research found that genome-wide methylation profiles could help accurately identify CA19-9-negative PAC cases [31]. In this study, we revealed that the fragmentomics-based stacked model achieved high sensitivity (18 out of 19, 94.7%) in differentiating biliopancreatic cancers from healthy volunteers in the CA19-9-negative group. Furthermore, after integrating the stacked model with CA19-9, the final CF model showed great performance in biliopancreatic cancers detection (AUC = 0.995). According to above, our study suggested that cfDNA fragmentomics could be an important complement to CA19-9 in biliopancreatic cancers detection, which paved the way for the development of new diagnostic strategies, especially for patients in the CA19-9-negative group.

Although our preliminary results of using cfDNA fragmentomics to facilitate non-invasive detection of biliopancreatic cancers were encouraging, several limitations should be acknowledged. The relatively small size of the study cohort might impair the performance of our model, which needs to be validated with a larger population. In addition, the sample sizes of several key categories such as stage I of PAC, CCA and GBC were also limited. Therefore, there might be overestimation of model sensitivity, and the model could need some optimization for early detection or screening purposes in biliopancreatic cancers. Finally, our models were constructed based only on cancer and healthy cases. Further inclusion of samples from benign biliopancreatic diseases would help improve the performance of our model in distinguishing biliopancreatic cancers patients and promote its clinical application.

## Conclusions

In summary, we reported a machine learning model based on fragmentomics features of plasma cfDNA for detecting biliopancreatic cancers. Our method relying on only low-coverage WGS data remained great performance in distinguishing biliopancreatic cancers and helped in differentiating subtypes. The final model that integrated cfDNA fragmentomics features with CA19-9 exhibited excellent clinical potential for its ultrasensitivity and provided an affordable way for improving cancer detection in clinical practice.

### Supplementary Information

Supplementary Material 1: Figure S1. Different motifs of the reads across samples. (A) Average GC content along the reads of our sequencing data. UMIs were added during sequencing libraries preparation. (B) Average GC content of several samples randomly selected in our dataset and sequenced without UMIs. (C-D) Average GC content of public dataset SRP262262 (C) and GSE71378 (D) sequenced without UMI. Note that GSE71378 is noisy because its data was sequenced with two different read lengths. (E-H) Top four motifs of public dataset SRP262262 (E), GSE71378 (F), our sequencing data with UMIs counted from 1^st^ bp (G) or from 6^th^ bp (H).

Supplementary Material 2: Figure S2. ROC curves of 4-bp or 6-bp end motif models. Performance of the 4-bp end motif model in the training cohort (A) and in the validation cohort (C). Performance of the 6-bp end motif in the training cohort (B) and in the validation cohort (D).

Supplementary Material 3: Figure S3. Performance of single-feature models trained on the downsized WGS data. ROC curves of the stacked model (A), fragment size model (B), the end motif model (C), and the NF model (D) using the downsized WGS data.

Supplementary Material 4: Figure S4. Differentiation of single biliopancreatic cancer types using cfDNA fragmentomics features. ROC curves of the models trained on all three features (A) or fragment size (B), end motif (C), and NF (D) feature alone to predict one cancer type against another.

Supplementary Material 5: Figure S5. Construction and evaluation of the LinearSVC classifier integrating the cancer scores of the stacked model and the log2-transformed CA19-9 values. (A-B) The classifiers were visualized as a single line (A) and the ROC curve (B) for all cancers in the training cohort. (C) The cancer scores calculated from the CF model for all patients in the validation cohort. (D) Box plots illustrating the distribution of cancer scores generated from the CF model in non-cancer individuals, stage I/II and stage III/IV patients of each cancer type. (E-M) Construction and evaluation of the CF model in CCA (E-G), GBC (H-J), and PAC (K-M) patients.

Supplementary Material 6: Table S1. Performance of models trained on single feature or stacked features in the training cohort.

Supplementary Material 7: Table S2. Performance of models trained on single feature or stacked features in the validation cohort.

Supplementary Material 8: Table S3. AUCs of 10-fold cross-validation on the training cohort.

Supplementary Material 9: Table S4. Performance of single feature models or the stacked model using down-sized WGS data in the validation cohort.

Supplementary Material 10: Table S5. Gene coefficients of the NF model.

Supplementary Material 11: Table S6. Enrichment analysis of the top 500 genes that contribute the most to predict PAC against CCA/GBC in the NF model using ToppGene.

Supplementary Material 12: Table S7. CF model performance.

## Data Availability

The datasets generated have been deposited into the Genome Sequence Archive for Human (GSA-Human) of China National Genomics Data Center (NGDC) under accession number PRJCA021459.

## Abbreviations

AUC: Area under curve
CA19-9: Carbohydrate antigen 19–9
cfDNA: Cell-free DNA
CCA: Cholangiocarcinoma
GBC: Gallbladder cancer
PAC: Pancreatic cancer
TSS: Transcription start site
TNM: Tumor-node-metastasis,
UMI: Unique molecular identifier

## References

[1] Mizrahi JD, Surana R, Valle JWShroff RT.  (2020). Pancreatic cancer. Lancet.

[2] Rocio IRM, Vincenzo C, Timothy JK, Matias AA, Maria G, Cedric C (2022). Clinical relevance of biomarkers in cholangiocarcinoma: critical revision and future directions. Gut.

[3] Gao Q, Zeng Q, Wang Z, Li C, Xu Y, Cui P (2022). Circulating cell-free DNA for cancer early detection. Innovation (Camb).

[4] Ignatiadis M, Sledge GWJeffrey SS.  (2021). Liquid biopsy enters the clinic — implementation issues and future challenges. Nat Rev Clin Oncol.

[5] Moss J, Magenheim J, Neiman D, Zemmour H, Loyfer N, Korach A (2018). Comprehensive human cell-type methylation atlas reveals origins of circulating cell-free DNA in health and disease. Nat Commun.

[6] Ulz P, Perakis S, Zhou Q, Moser T, Belic J, Lazzeri I (2019). Inference of transcription factor binding from cell-free DNA enables tumor subtype prediction and early detection. Nat Commun.

[7] Song P, Wu LR, Yan YH, Zhang JX, Chu T, Kwong LN (2022). Limitations and opportunities of technologies for the analysis of cell-free DNA in cancer diagnostics. Nature Biomedical Engineering.

[8] Lo YMD, Han DSC, Jiang P, Chiu RWK (2021). Epigenetics, fragmentomics, and topology of cell-free DNA in liquid biopsies. Science.

[9] Cristiano S, Leal A, Phallen J, Fiksel J, Adleff V, Bruhm DC (2019). Genome-wide cell-free DNA fragmentation in patients with cancer. Nature.

[10] Mouliere F, Chandrananda D, Piskorz AM, Moore EK, Morris J, Ahlborn LB et al. Enhanced detection of circulating tumor DNA by fragment size analysis. Sci Transl Med. 2018;10:eaat4921. 10.1126/scitranslmed.aat4921 PMC6483061 30404863

[11] Jiang P, Sun K, Tong YK, Cheng SH, Cheng THT, Heung MMS (2018). Preferred end coordinates and somatic variants as signatures of circulating tumor DNA associated with hepatocellular carcinoma. Proc Natl Acad Sci U S A.

[12] Snyder MW, Kircher M, Hill AJ, Daza RMShendure J.  (2016). Cell-free DNA Comprises an In Vivo Nucleosome Footprint that Informs Its Tissues-Of-Origin. Cell.

[13] Chen L, Abou-Alfa GK, Zheng B, Liu JF, Bai J, Du LT (2021). Genome-scale profiling of circulating cell-free DNA signatures for early detection of hepatocellular carcinoma in cirrhotic patients. Cell Res.

[14] Ma X, Chen Y, Tang W, Bao H, Mo S, Liu R (2021). Multi-dimensional fragmentomic assay for ultrasensitive early detection of colorectal advanced adenoma and adenocarcinoma. J Hematol Oncol.

[15] Zhang X, Wang Z, Tang W, Wang X, Liu R, Bao H (2022). Ultrasensitive and affordable assay for early detection of primary liver cancer using plasma cell-free DNA fragmentomics. Hepatology.

[16] Bao H, Wang Z, Ma X, Guo W, Zhang X, Tang W (2022). Letter to the Editor: An ultra-sensitive assay using cell-free DNA fragmentomics for multi-cancer early detection. Mol Cancer.

[17] Oruba A, Essen SS, D.  (2020). Role of cell-type specific nucleosome positioning in inducible activation of mammalian promoters. Nat Commun.

[18] Lo YM, Chan KC, Sun H, Chen EZ, Jiang P, Lun FM (2010). Maternal plasma DNA sequencing reveals the genome-wide genetic and mutational profile of the fetus. Sci Transl Med.

[19] Jiang P, Chan CW, Chan KC, Cheng SH, Wong J, Wong VW (2015). Lengthening and shortening of plasma DNA in hepatocellular carcinoma patients. Proc Natl Acad Sci U S A.

[20] Jiang P, Sun K, Peng W, Cheng SH, Ni M, Yeung PC (2020). Plasma DNA End-Motif Profiling as a Fragmentomic Marker in Cancer, Pregnancy, and Transplantation. Cancer Discov.

[21] Ulz P, Thallinger GG, Auer M, Graf R, Kashofer K, Jahn SW (2016). Inferring expressed genes by whole-genome sequencing of plasma DNA. Nat Genet.

[22] Ardlie KG, Deluca DS, Segrè AV, Sullivan TJ, Young TR, The GC (2015). The Genotype-Tissue Expression (GTEx) pilot analysis: Multitissue gene regulation in humans. Science.

[23] Han BW, Yang X, Qu SF, Guo ZW, Huang LM, Li K (2021). A Deep-Learning Pipeline for TSS Coverage Imputation From Shallow Cell-Free DNA Sequencing. Front Med (Lausanne).

[24] Chen J, Bardes EE, Aronow BJJegga AG.  (2009). ToppGene Suite for gene list enrichment analysis and candidate gene prioritization. Nucleic Acids Res.

[25] Munakata Y, Yamada T, Imai J, Takahashi K, Tsukita S, Shirai Y (2018). Olfactory receptors are expressed in pancreatic beta-cells and promote glucose-stimulated insulin secretion. Sci Rep.

[26] Wei P, Tang HLi D.  (2012). Insights into pancreatic cancer etiology from pathway analysis of genome-wide association study data. PLoS ONE.

[27] Goonetilleke KSSiriwardena AK.  (2007). Systematic review of carbohydrate antigen (CA 19–9) as a biochemical marker in the diagnosis of pancreatic cancer. Eur J Surg Oncol.

[28] Moser T, Kuhberger S, Lazzeri I, Vlachos GHeitzer E.  (2023). Bridging biological cfDNA features and machine learning approaches. Trends Genet.

[29] Guo W, Chen X, Liu R, Liang N, Ma Q, Bao H (2022). Sensitive detection of stage I lung adenocarcinoma using plasma cell-free DNA breakpoint motif profiling. EBioMedicine.

[30] Guo S, Shi X, Shen J, Gao S, Wang H, Shen S (2020). Preoperative detection of KRAS G12D mutation in ctDNA is a powerful predictor for early recurrence of resectable PDAC patients. Br J Cancer.

[31] Wu H, Guo S, Liu X, Li Y, Su Z, He Q (2022). Noninvasive detection of pancreatic ductal adenocarcinoma using the methylation signature of circulating tumour DNA. BMC Med.

[32] Ptashkin RN, Mandelker DL, Coombs CC, Bolton K, Yelskaya Z, Hyman DM (2018). Prevalence of Clonal Hematopoiesis Mutations in Tumor-Only Clinical Genomic Profiling of Solid Tumors. JAMA Oncol.

[33] Ben-Ami R, Wang QL, Zhang J, Supplee JG, Fahrmann JF, Lehmann-Werman R (2024). Protein biomarkers and alternatively methylated cell-free DNA detect early stage pancreatic cancer. Gut.

[34] Hartwig C, Muller J, Klett H, Kouhestani D, Mittelstadt A, Anthuber A (2024). Discrimination of pancreato-biliary cancer and pancreatitis patients by non-invasive liquid biopsy. Mol Cancer.

[35] Lapin M, Oltedal S, Tjensvoll K, Buhl T, Smaaland R, Garresori H (2018). Fragment size and level of cell-free DNA provide prognostic information in patients with advanced pancreatic cancer. J Transl Med.

[36] Wintachai P, Lim JQ, Techasen A, Lert-Itthiporn W, Kongpetch S, Loilome W et al. Diagnostic and Prognostic Value of Circulating Cell-Free DNA for Cholangiocarcinoma. Diagnostics (Basel). 2021;11:999. 10.3390/diagnostics11060999 PMC8228499 34070951

[37] Kumari S, Tewari S, Husain N, Agarwal A, Pandey A, Singhal A (2017). Quantification of Circulating Free DNA as a Diagnostic Marker in Gall Bladder Cancer. Pathol Oncol Res.

[38] Qi T, Pan M, Shi H, Wang L, Bai YGe Q. Cell-Free DNA Fragmentomics: The Novel Promising Biomarker. Int J Mol Sci. 2023;24:1503. 10.3390/ijms24021503 PMC9866579 36675018

[39] Maßberg DHatt H.  (2018). Human Olfactory Receptors: Novel Cellular Functions Outside of the Nose. Physiol Rev.

[40] Jones S, Zhang X, Parsons DW, Lin JC, Leary RJ, Angenendt P (2008). Core signaling pathways in human pancreatic cancers revealed by global genomic analyses. Science.

[41] Lai Y, Zhang F, Nayak TK, Modarres R, Lee NHMcCaffrey TA.  (2017). Detecting discordance enrichment among a series of two-sample genome-wide expression data sets. BMC Genomics.

[42] Cao T, Wu HJi T.  (2023). Bioinformatics-based construction of prognosis-related methylation prediction model for pancreatic cancer patients and its application value. Front Pharmacol.

[43] Luo G, Jin K, Deng S, Cheng H, Fan Z, Gong Y (2021). Roles of CA19-9 in pancreatic cancer: Biomarker, predictor and promoter. Biochim Biophys Acta Rev Cancer.

[44] Luo G, Liu C, Guo M, Cheng H, Lu Y, Jin K (2017). Potential Biomarkers in Lewis Negative Patients With Pancreatic Cancer. Ann Surg.

