# Supplementary material for: Detection and characterization of pancreatic and biliary tract cancers using cell-free DNA fragmentomics
Source: J Exp Clin Cancer Res. 2024 May 15;43:145. doi: 10.1186/s13046-024-03067-y (PMC11094938; doi:10.1186/s13046-024-03067-y)
Supplement: Supplementary file 1 — Supplementary Material 1: Figure S1. Different motifs of the reads across samples. (A) Average GC content along the reads of our sequencing data. UMIs were added during sequencing libraries preparation. (B) Average GC content of several samples randomly selected in our dataset and sequenced without UMIs. (C-D) Average GC content of public dataset SRP262262 (C) and GSE71378 (D) sequenced without UMI. Note that GSE71378 is noisy because its data was sequenced with two different read lengths. (E-H) Top four motifs of public dataset SRP262262 (E), GSE71378 (F), our sequencing data with UMIs counted from 1st bp (G) or from 6th bp (H). [file 13046_2024_3067_MOESM1_ESM.pdf]

Figure S1

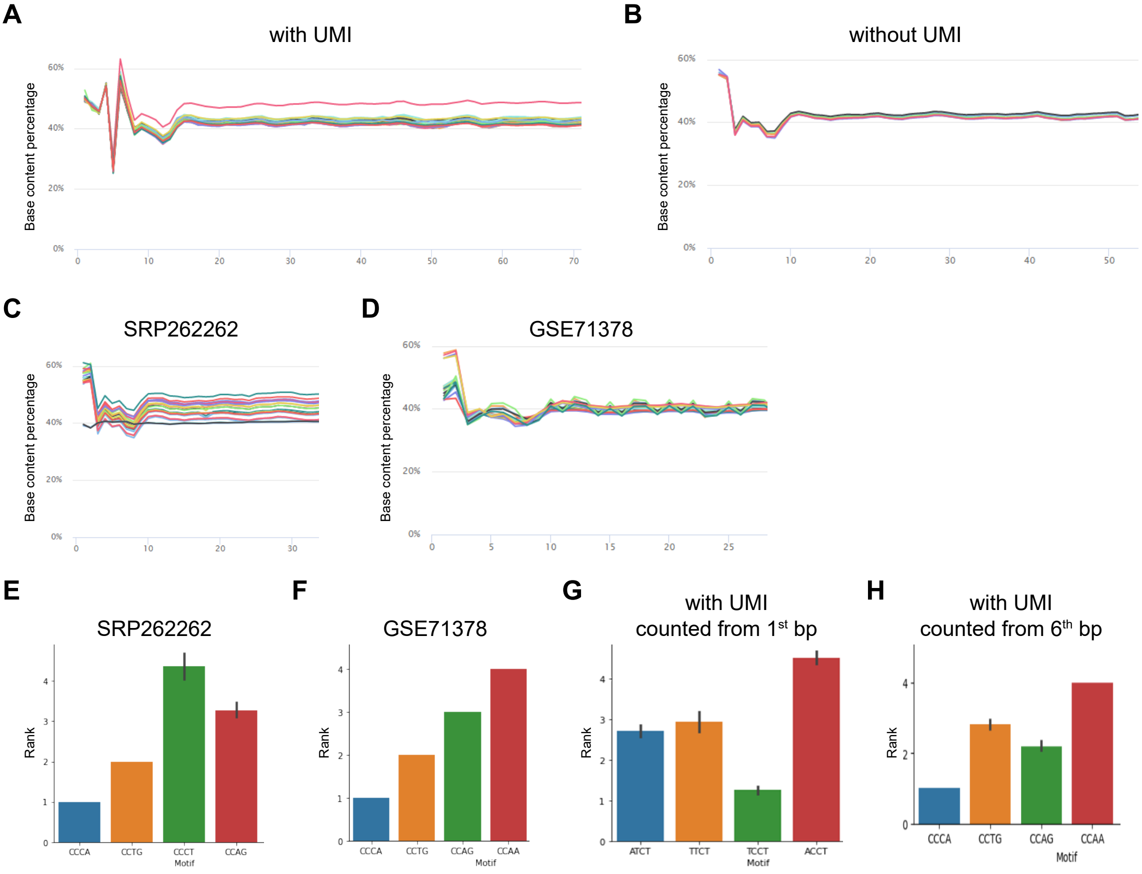

A
with UMI
Base content percentage
60%
40%
20%
0%
0
10
20
30
40
50
60
70
B
without UMI
Base content percentage
60%
40%
20%
0%
0
10
20
30
40
50
C
SRP262262
Base content percentage
60%
40%
20%
0%
0
10
20
30
D
GSE71378
Base content percentage
60%
40%
20%
0%
0
5
10
15
20
25
E
SRP262262
Rank
CCCA
CCTG
CCCT
CCAG
Motif
F
GSE71378
Rank
CCCA
CCTG
CCAG
CCAA
Motif
G
with UMI
counted from 1st bp
Rank
ATCT
TTCT
TCCT
ACCT
Motif
H
with UMI
counted from 6th bp
Rank
CCCA
CCTG
CCAG
CCAA
Motif
