# Supplementary figures and images for: Detection and characterization of pancreatic and biliary tract cancers using cell-free DNA fragmentomics
Source: J Exp Clin Cancer Res. 2024 May 15;43:145. doi: 10.1186/s13046-024-03067-y (PMC11094938; doi:10.1186/s13046-024-03067-y)

**Figure S2**

**A**

Motif - 4bp

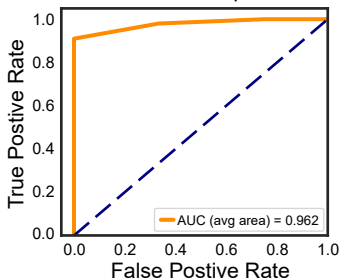

**B**

Motif - 6bp

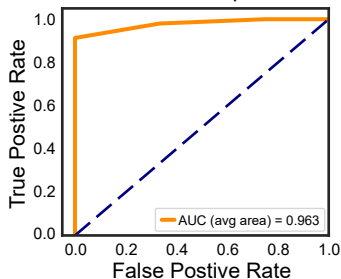

**C**

Validation

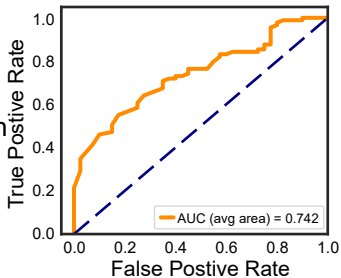

**D**

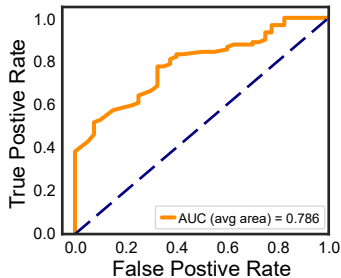

Supplement: Supplementary file 2 — Supplementary Material 2: Figure S2. ROC curves of 4-bp or 6-bp end motif models. Performance of the 4-bp end motif model in the training cohort (A) and in the validation cohort (C). Performance of the 6-bp end motif in the training cohort (B) and in the validation cohort (D). [file 13046_2024_3067_MOESM2_ESM.pdf]

**Figure S3****A**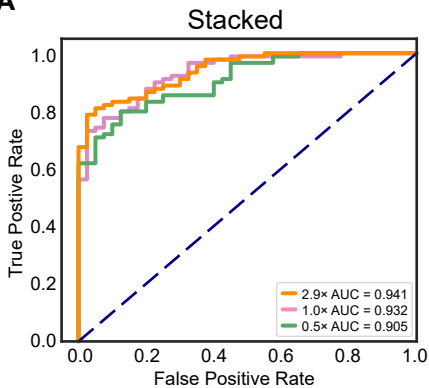**B**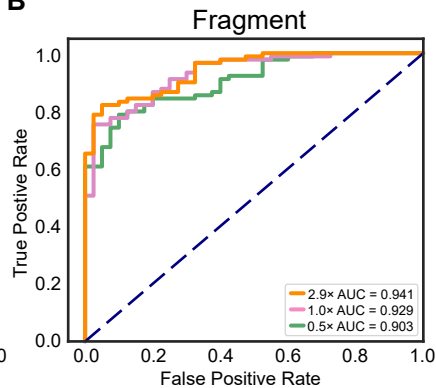**C**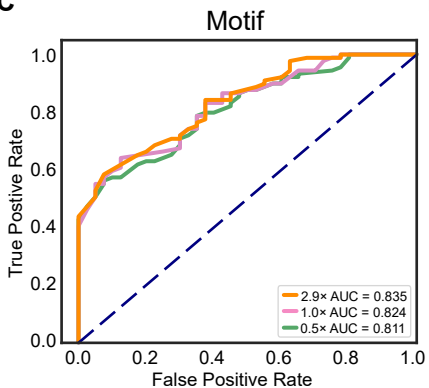**D**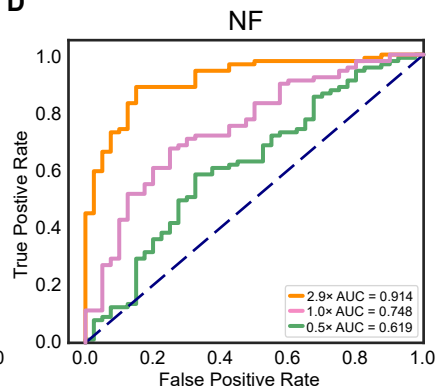

Supplement: Supplementary file 3 — Supplementary Material 3: Figure S3. Performance of single-feature models trained on the downsized WGS data. ROC curves of the stacked model (A), fragment size model (B), the end motif model (C), and the NF model (D) using the downsized WGS data. [file 13046_2024_3067_MOESM3_ESM.pdf]

**Figure S4****A**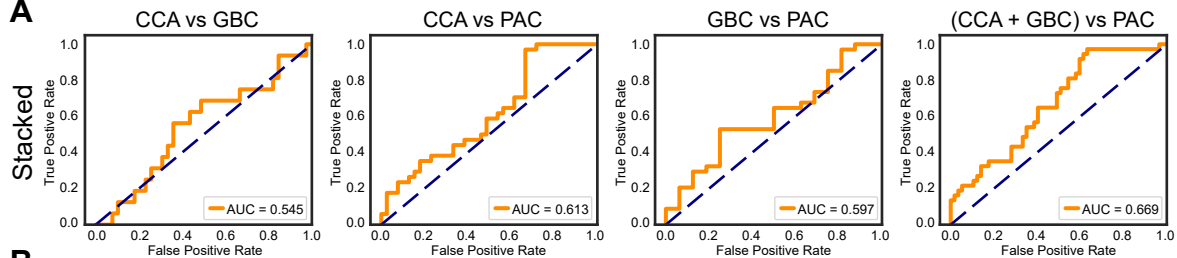**B**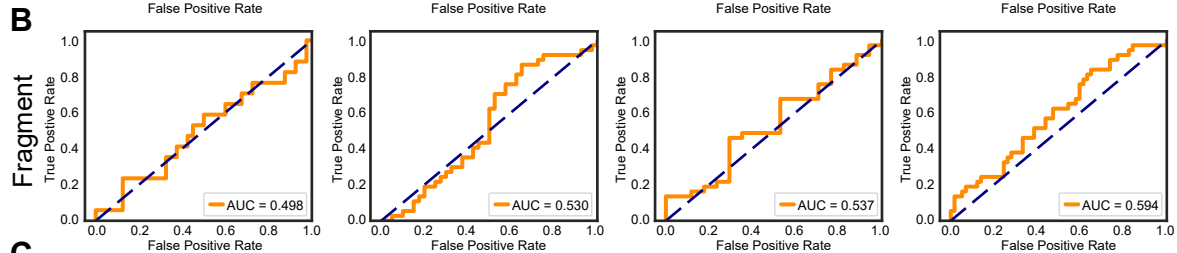**C**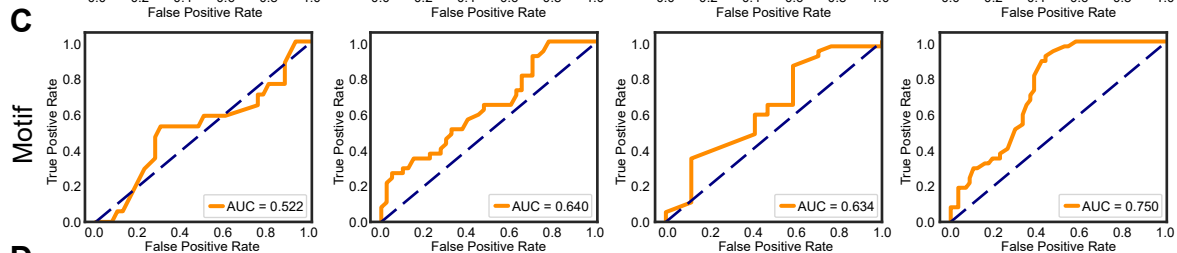**D**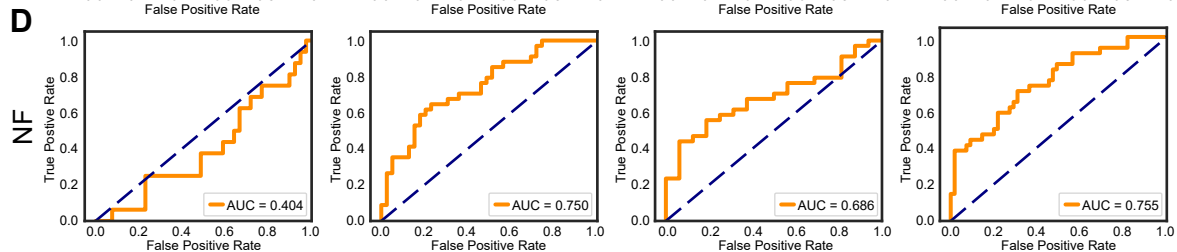

Supplement: Supplementary file 4 — Supplementary Material 4: Figure S4. Differentiation of single biliopancreatic cancer types using cfDNA fragmentomics features. ROC curves of the models trained on all three features (A) or fragment size (B), end motif (C), and NF (D) feature alone to predict one cancer type against another. [file 13046_2024_3067_MOESM4_ESM.pdf]

**Figure S5**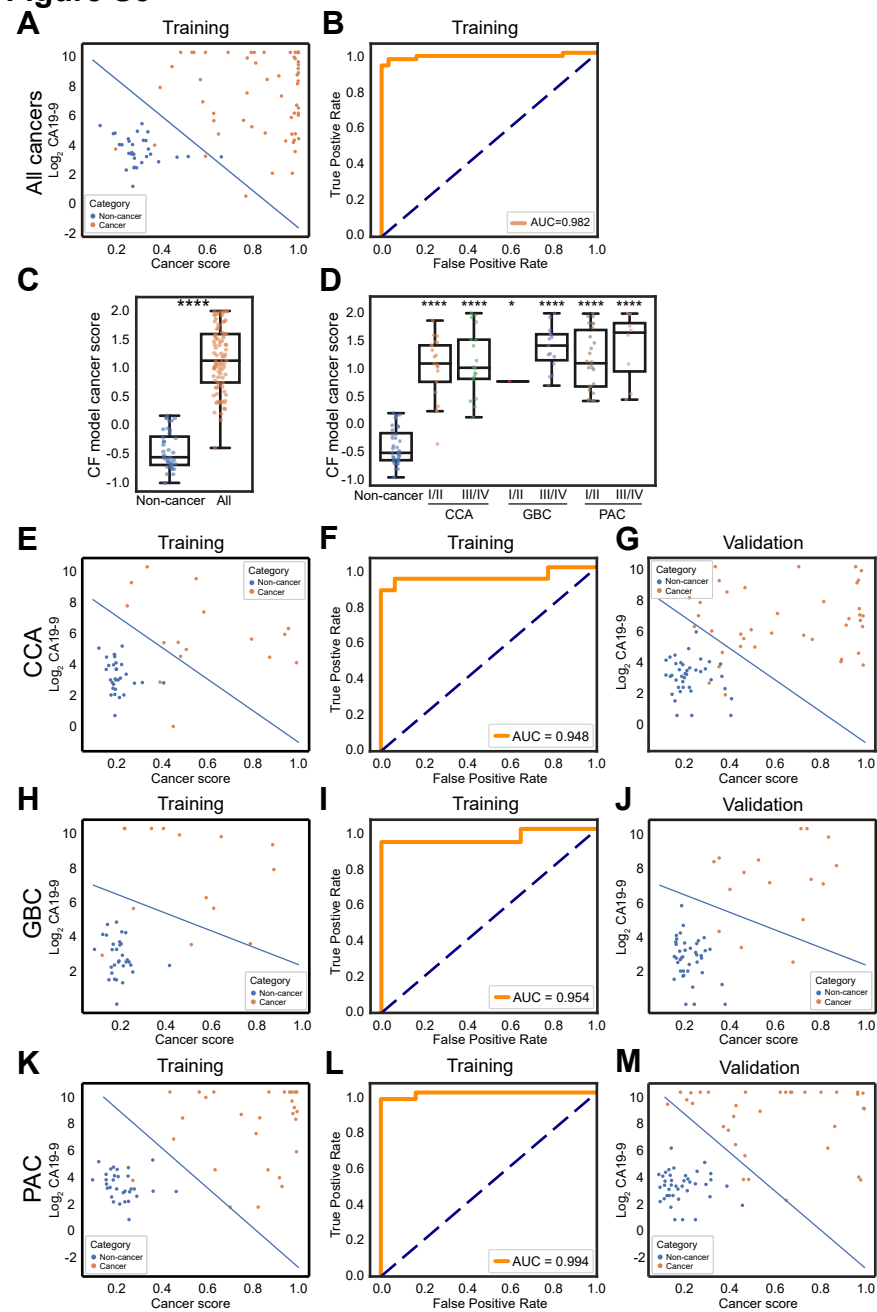

Supplement: Supplementary file 5 — Supplementary Material 5: Figure S5. Construction and evaluation of the LinearSVC classifier integrating the cancer scores of the stacked model and the log2-transformed CA19-9 values. (A-B) The classifiers were visualized as a single line (A) and the ROC curve (B) for all cancers in the training cohort. (C) The cancer scores calculated from the CF model for all patients in the validation cohort. (D) Box plots illustrating the distribution of cancer scores generated from the CF model in non-cancer individuals, stage I/II and stage III/IV patients of each cancer type. (E-M) Construction and evaluation of the CF model in CCA (E-G), GBC (H-J), and PAC (K-M) patients. [file 13046_2024_3067_MOESM5_ESM.pdf]
